# Florid cutaneous morbilliform eruption in the setting of primary Epstein-Barr virus infection

**DOI:** 10.1016/j.jdcr.2023.11.031

**Published:** 2024-01-29

**Authors:** Shahin A. Saberi, Anusha M. Kumar, Dale Davis, Vinod E. Nambudiri

**Affiliations:** a Harvard Medical School, Boston, Massachusetts; b Department of Dermatology, Brigham and Women's Hospital, Boston, Massachusetts; c Department of Pathology, Brigham and Women's Hospital, Boston, Massachusetts

**Keywords:** cutaneous hypersensitivity, dermatopathology, Epstein-Barr virus, inflammatory disease, morbilliform eruption

## Introduction

Epstein-Barr virus (EBV), a member of the *Herpesviridae* family, is a highly prevalent virus with a range of clinical presentations from asymptomatic infection to infectious mononucleosis (IM). IM occurs in a subset of primary EBV infections as a clinical syndrome typically involving fever, malaise, lymphadenopathy, and mucosal inflammation (tonsillitis and pharyngitis) with associated sore throat. In patients with EBV IM, generalized cutaneous manifestations can include persistent morbilliform eruptions in patients exposed to antibiotics or more subtle, transient morbilliform eruptions in up to 15% of unexposed patients—though other morphologies (eg, papulovesicular, petechial) can arise infrequently.^1^, ^2^, ^3^ Generalized cutaneous manifestations are primarily associated with EBV IM, not milder forms of EBV infections in adults. Herein, we present a patient with a florid cutaneous hypersensitivity reaction in the setting of an otherwise low-grade primary EBV infection and no other identified triggers.

## Case Report

**Fig 1:**
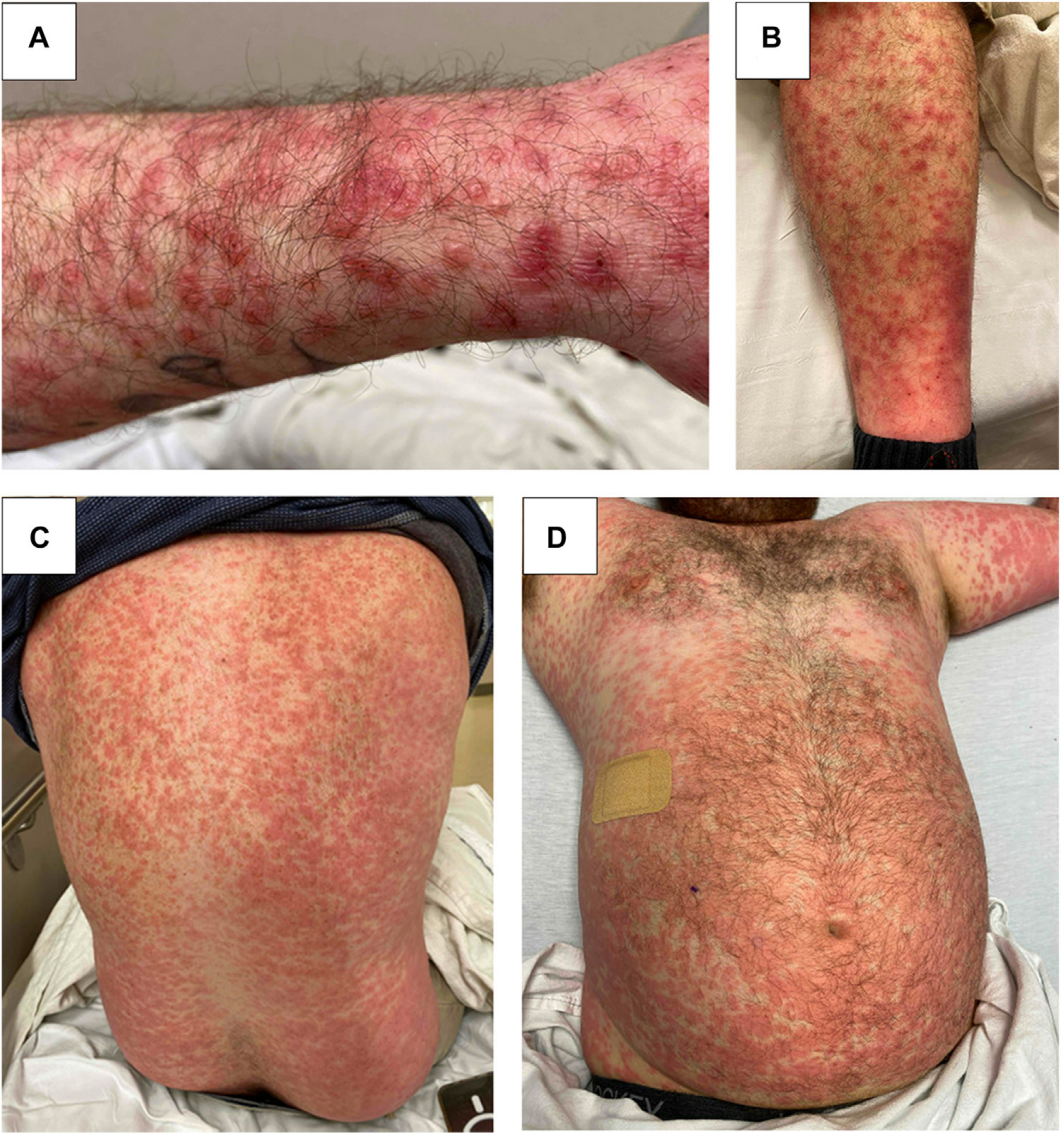
Generalized erythematous papules coalescing into thin plaques diffusely with overlying vesicles on the forearm (**A**), shin (**B**), back (**C**), and abdomen (**D**). Biopsies were taken from vesiculated papules on the forearm in the panel (**A**).

A 37-year-old male without significant past medical history was admitted with a diffuse cutaneous eruption. Erythematous, pink-red papules coalescing into thin plaques appeared first on the dorsal hands, progressively spreading to involve the majority of his body surface area within days. Follicular accentuation was noted, and the eruption began to vesiculate on the bilateral forearms and abdomen. Notably, the papulovesicular eruption spared the palms, soles, and mucous membranes; the palms only demonstrated mild diffuse erythema (Fig 1). The patient described the rash as pruritic but not painful or burning. Aside from intermittent low-grade fevers and subjective chills, the patient was asymptomatic. The patient denied sore throat, cough, recent prescription or over-the-counter medication exposures, or any sick contacts. He reported chronic nicotine use and recent marijuana use.

A complete blood count was notable for a leukocytosis (white blood cell count of 14.4 K/μL) with neutrophilic predominance (86.1%). The patient's liver and kidney function tests were within normal limits. His C-reactive protein level was markedly elevated at 145.7 mg/L, while his erythrocyte sedimentation rate was normal at 18 mm/h.

A broad infectious workup was completed. Blood and urine cultures did not grow any pathogens.

Testing for influenza A and B, SARS-CoV 2, respiratory syncytial virus, parainfluenza virus, adenovirus, human metapneumovirus, rhinovirus, parvovirus B19, human immunodeficiency virus 1 and 2, *Treponema*, *Babesia*, *Rickettsia*, *Ehrlichia*, *Anaplasma*, Lyme, tularemia, *Chlamydia*, *Gonorrhea*, *Strep pyogenes*, and *Mycoplasma* indicated no infection or prior immunity. Serology for EBV demonstrated positive viral capsid antigen IgM, negative viral capsid antigen IgG, and negative EBV nuclear antigen IgG, consistent with primary EBV infection.

**Fig 2:**
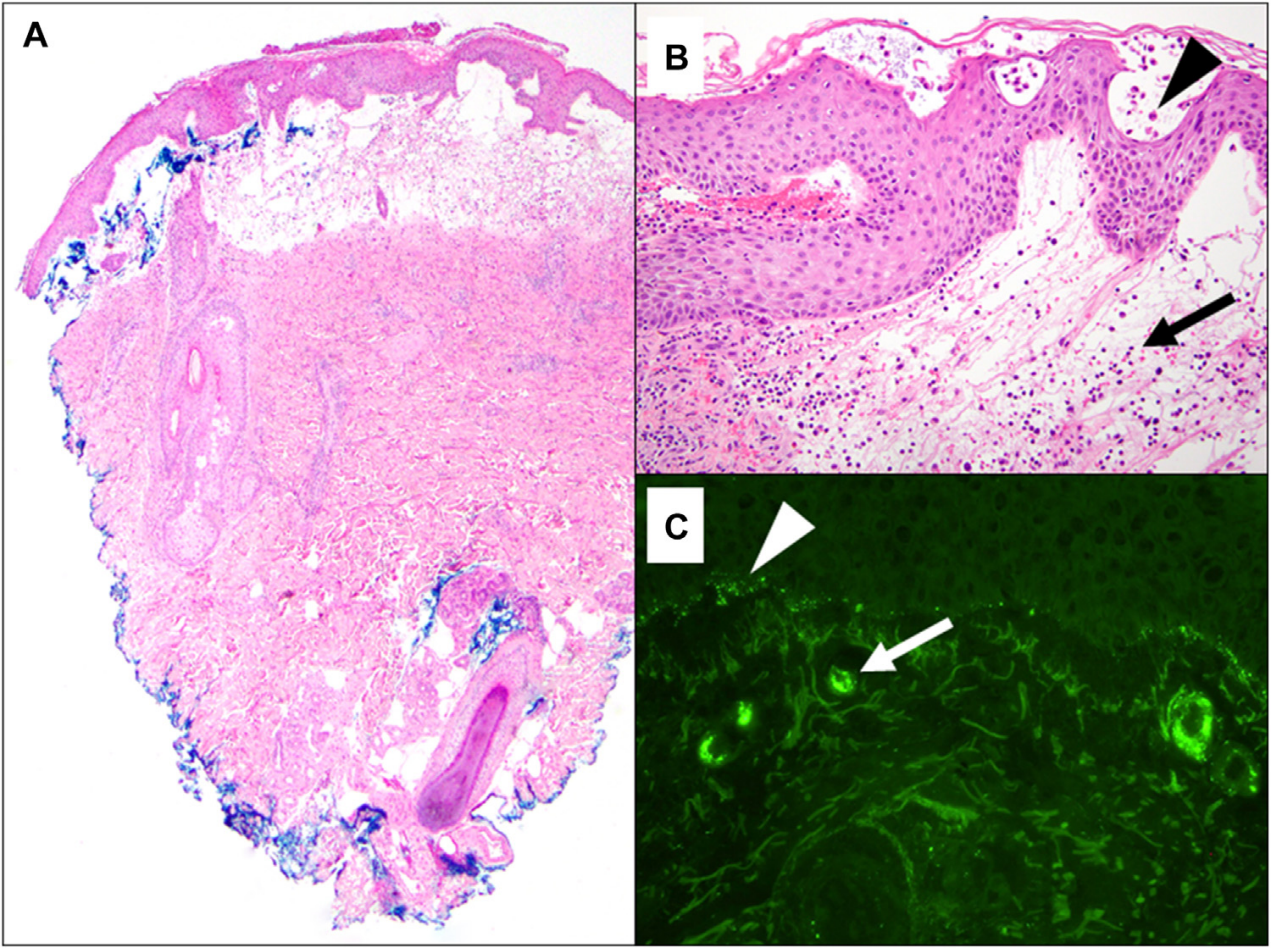
Biopsy findings. Representative photomicrograph of hematoxylin &and eosin–stained sections from punch biopsy collected from the left forearm (**A**). Prominent papillary dermal vesicle formation is apparent at scanning magnification. Investigation at higher power reveals acute spongiotic dermatitis with subcorneal and intradermal vesicles, which contain a mixed inflammatory infiltrate of neutrophils (*arrowhead*), lymphocytes, histiocytes, and rare eosinophils as well as extravasated erythrocytes (*arrow*). Parakeratosis and a dermal perivascular lymphocytic infiltrate are present (**B**). Direct immunofluorescence study showing 3+ C3 granular deposits at the dermal-epidermal junction (*arrowhead*) and nonspecific staining at the superficial vessels (*arrow*) (**C**).

While the infectious workup was pending, 2 4-mm punch biopsies were performed on the left forearm for hematoxylin and eosin staining and direct immunofluorescence. Biopsy samples demonstrated acute spongiotic dermatitis with subcorneal and intradermal vesicle formation, and mixed inflammatory infiltrate comprising rare eosinophils, neutrophils, and perivascular lymphohistiocytic inflammation on hematoxylin and eosin staining, as well as 3-to-4+ C3 and trace-to-1+ granular IgA and IgM localized to the walls of superficial microvessels on direct immunofluorescence (Fig 2). Epstein-Barr encoding region in situ hybridization was negative on the skin biopsies.

The patient received 1 125-mg dose of methylprednisolone intravenously followed by 60 mg of oral prednisone daily for 3 days, tapered by 10 mg every 3 days thereafter. Adjunctive treatment included fexofenadine 180 mg daily and topical triamcinolone 0.1% ointment as needed to the affected areas. Significant improvement in lesions was noted within 5 days of treatment, with near-complete resolution noted at an 8-week follow-up visit.

## Discussion

We present a case of a diffuse, pseudo-vesiculated morbilliform eruption in the setting of primary EBV infection without antibiotic exposure or evidence of IM. Other than intermittent fevers, the patient presented with no localizing symptoms.

Rarely, cutaneous lesions are the presenting sign of otherwise asymptomatic primary EBV infection—prior cases include a 20-month-old girl with Giannotti-Crosti syndrome^4^ and a 43-year-old male with a pityriasis lichenoides-like eruption.^5^ An underrecognized but striking presenting sign of primary EBV is vulvar ulceration.^6^ Additional hypersensitivity responses have been reported in children, including erythema nodosum and acute generalized exanthematous pustulosis.^7^ However, a florid cutaneous hypersensitivity reaction-like eruption as the presenting sign of otherwise low-grade EBV infection has not previously been reported in adults.

The lack of atypical lymphocytes and negative Epstein-Barr encoding region in situ hybridization on biopsy suggests that our patient's cutaneous eruption was an inflammatory hypersensitivity response to EBV rather than the result of direct EBV infection of skin cells. EBV can induce a robust cytotoxic T-cell immune response, which may have led to a T-cell–mediated cutaneous hypersensitivity reaction in this patient. The patient's use of marijuana in the days preceding the onset of the rash may also be implicated. In patients with EBV infection, antibiotic-induced rashes are thought to result from transient alterations of the immune system which enhance the immune response to drugs and lead to delayed hypersensitivity reactions.^8^^,^^9^ Given the papulovesicular morphology of lesions, adult Giannotti-Crosti syndrome was considered, but we favored characterizing this presentation as a florid hypersensitivity given the prominent truncal involvement, mixed infiltrate on histology, and demographics of the patient—Giannotti-Crosti syndrome is classically an acrally-distributed pediatric eruption that may rarely occur in the adult population.^10^

This case demonstrates that a primary EBV infection can trigger a florid morbilliform eruption even in the absence of antibiotic exposure and expands our understanding of cutaneous manifestations of acute EBV. We are also reminded that in the context of clinical and/or pathological evidence of a cutaneous hypersensitivity reaction, a broad infectious workup is warranted when an obvious medication or alternate culprit is not identified.

## Conflicts of interest

None disclosed.
